# LAA occlusion is effective and safe in very high-risk atrial fibrillation patients with prior stroke: results from the multicentre German LAARGE registry

**DOI:** 10.1007/s00392-024-02376-8

**Published:** 2024-01-31

**Authors:** Uzair Ansari, Johannes Brachmann, Thorsten Lewalter, Uwe Zeymer, Horst Sievert, Jakob Ledwoch, Volker Geist, Matthias Hochadel, Steffen Schneider, Jochen Senges, Ibrahim Akin, Christian Fastner

**Affiliations:** 1 Department of Cardiology, Angiology, Haemostaseology and Medical Intensive Care, University Medical Centre Mannheim (UMM), Medical Faculty Mannheim, Heidelberg University, European Center for AngioScience (ECAS), and German Center for Cardiovascular Research (DZHK) Partner Site Heidelberg/Mannheim, Theodor-Kutzer-Ufer 1-3, 68167 Mannheim, Germany; 2 Department of Cardiology, Angiology, and Pneumology, Second Medical Clinic, Coburg Hospital, Coburg, Germany; 3 University of Split School of Medicine, Split, Croatia; 4 Department of Medicine, Cardiology, and Intensive Care, Hospital Munich-Thalkirchen, Munich, Germany; 5 Medizinische Klinik B, Klinikum Ludwigshafen, Ludwigshafen am Rhein, Germany; 6 CardioVascular Center (CVC) Frankfurt, Frankfurt am Main, Germany; 7 Isar Herz Zentrum München, ISAR Klinikum, Munich, Germany; 8 Klinik für Kardiologie, Pneumologie und Internistische Intensivmedizin, München Klinik Neuperlach, Munich, Germany; 9 Department of Cardiology, Heart Center, Segeberger Kliniken, Bad Segeberg, Germany; 10 Stiftung Institut für Herzinfarktforschung, Ludwigshafen am Rhein, Germany

**Keywords:** Bleeding events, Ischemic stroke, LAARGE, Left atrial appendage closure, Nonvalvular atrial fibrillation, Thromboembolism

## Abstract

**Background:**

Interventional left atrial appendage occlusion (LAAO) mitigates the risk of thromboembolic events in nonvalvular atrial fibrillation (AF) patients with contraindication for long-term oral anticoagulation (OAC). Patients with prior stroke have a relevantly increased risk of recurrent stroke, so the effectiveness of LAAO could be reduced in this specific very high-risk patient group.

**Aim:**

This sub-study of the LAARGE registry investigates the effectiveness and safety of LAAO for secondary prevention in nonvalvular AF patients with a history of stroke.

**Methods:**

LAARGE is a prospective, non-randomised registry on the clinical reality of LAAO. The current sub-study employs data from index procedure and 1-year follow-up. Effectiveness and safety were assessed by documentation of all-cause mortality, non-fatal thromboembolism, procedure-related complications, and bleeding events.

**Results:**

A total of 638 patients were consecutively included from 38 hospitals in Germany and divided into two groups: 137 patients with a history of stroke (21.5%) and 501 patients without. Successful implantation was consistent between both groups (98.5% vs. 97.4%, *p* = NS), while peri-procedural MACCE and other complications were rare (0% vs. 0.6% and 4.4% vs. 4.0%, respectively; each *p* = NS). Kaplan–Meier estimate showed no significant difference in primary effectiveness outcome measure (freedom from all-cause death or non-fatal stroke) between both groups at follow-up (87.8% vs. 87.7%, *p* = NS). The incidence of transient ischemic attack or systemic embolism at follow-up was low (0% vs. 0.5% and 0.9% vs. 0%, respectively; each *p* = NS). Severe bleeding events after hospital discharge were rare (0% vs. 0.7%, *p* = NS).

**Conclusions:**

Patients with prior stroke demonstrated similar effectiveness and safety profile for LAAO as compared to patients without prior stroke. LAAO could serve as a feasible alternative to OAC for secondary stroke prevention in this selected group of nonvalvular AF patients.

**ClinicalTrials.gov identifier:**

NCT02230748.

**Graphical Abstract:**

**Figure:**
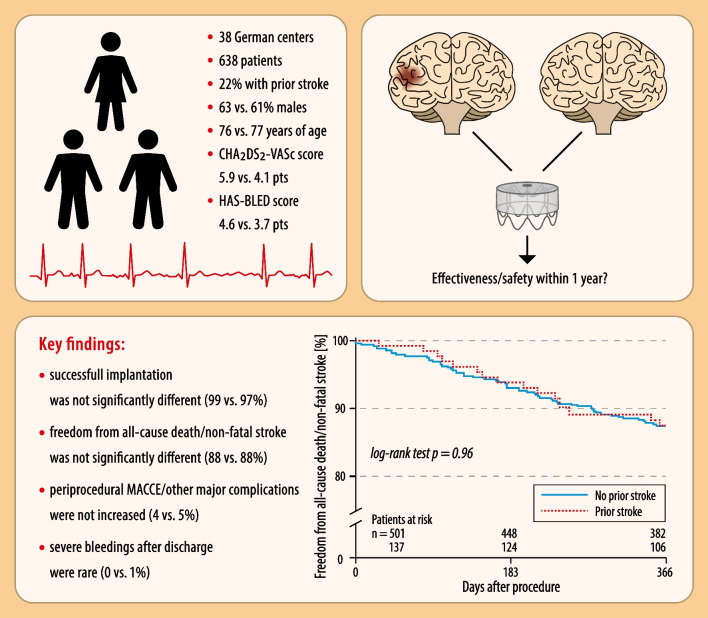
LAA occlusion in patients with and without prior stroke – results from the multicentre German LAARGE registry

**Supplementary Information:**

The online version contains supplementary material available at 10.1007/s00392-024-02376-8.

## Introduction

Atrial fibrillation (AF) is a common cardiac arrhythmia with an age-dependent prevalence of 2–4% in the adult population, and cerebrovascular stroke is perhaps the most serious complication of AF. Almost 20–30% of all ischemic strokes are associated with AF, and these cardioembolic events contribute to a relevant increase in mortality and morbidity [1].

Cerebrovascular stroke has a tendency to recur in AF patients [1, 2]. This could possibly be attributed to the fact that up to 20% of mostly high-risk patients (CHA_2_DS_2_-VASc score > 3 points) are not adequately protected due to poor dosing or have contraindications for therapeutic anticoagulation [3, 4]. Although the use of therapeutic anticoagulation has shown to be effective in reducing the risk of annual recurrent ischemic stroke, it has been estimated that oral anticoagulation (OAC) is prescribed for ischemic stroke prevention to only 15–44% of patients with AF and prior stroke due to its limitations [5]. Additionally, a Swedish registry showed that almost 55% of patients discontinued therapy with warfarin for secondary stroke prevention after 2 years due to associated complications [3].

Currently, the lack of robust data has meant that there is no clear consensus regarding secondary stroke prevention. OAC initiation/resumption after a cerebrovascular incident is dependent on multiple determinants including index event severity and infarct size [1, 6]. It is especially prudent to dissect the practice of therapeutic anticoagulation in these patients, as factors such as choice of OAC, adherence to therapy, and therapeutic range optimization also play a vital role in clinical decision-making [7, 8]. Additionally, as the affected age group is quite elderly, the choice of an age-appropriate OAC is extremely relevant. Ischemic stroke associated with AF in the elderly is usually severe, and many patients have residual disability and handicap with poor prognosis [9–11].

Early randomised controlled trials (RCTs) such as PROTECT-AF and PREVAIL could show that the percutaneous left atrial appendage occlusion (LAAO) was non-inferior to long-term warfarin therapy in nonvalvular AF patients. The PRAGUE-17 trial could show non-inferiority for LAAO compared to non-vitamin K antagonist OAC (NOAC). [12] Additionally, observational data has suggested its use in patients with contraindications to therapeutic OAC [7]. However, as the risk of suffering a stroke in patients with a prior stroke is significantly higher than in patients without a prior stroke [2], the effectiveness of LAAO in this very high-risk patient group might be different. In the PRAGUE-17 trial, only about one-third of patients in the LAAO group had suffered a prior stroke; the same was the case in the EWOLUTION registry [13]. A specific sub-group analysis of these patients was not performed. In the absence of data from RCTs, the benefit of LAAO in this patient group remains under debate. Evidence of high effectiveness in the targeted patient population is required to justify an invasive procedure to the patient. Until data from RCTs are available, specific data from large registries such as the Left Atrial Appendage Occluder Registry—GErmany (LAARGE; ClinicalTrials.gov Identifier: NCT02230748) are urgently needed to guide the clinical practice of secondary stroke prevention in this very high-risk population whenever long-term OAC is contraindicated. The aim of this sub-study was to investigate the LAARGE population for any potential benefits of using LAAO for the prevention of recurrent stroke whilst accounting for procedural safety and adverse events.

## Materials and methods

### Patient enrolment

LAARGE was designed as a prospective non-randomised multi-centre real-world registry recruiting eligible patients with LAAO intervention [14]. A total of 38 German centresers participated in this study conducted by the Institut für Herzinfarktforschung (IHF; Ludwigshafen am Rhein, Germany), an industry-independent sponsor, between the period of July 2014 and January 2016. The patients enrolled in this study represented a consecutively recruited population in whom the clinical diagnoses and treatment strategies were defined according to currently recommended guidelines to avoid any selection bias. The study was conducted in accordance with the Helsinki Declaration and was approved by the ethics committee of the Landesärztekammer Rheinland-Pfalz (Mainz, Germany). A written informed consent was obtained from all patients before study enrollment.

### Implantation procedure

Eligible patients diagnosed with non-valvular AF received at least a clinical examination, electrocardiogram, laboratory workup, and echocardiography/trans-oesophageal echocardiography (TOE). The pre-procedural screening and technical feasibility of LAAO was attested by the operating physician (varying levels of experience of each physician; < 10 to > 100 prior implantations). Standard devices certified for clinical routine were used [15]. The employed procedural technique was in line with recommendations set out by the European Heart Rhythm Association [16] or manufacturer. TOE or intracardiac echocardiography was used to rule out cardiac thrombi during the procedure. Moreover, it aided in transseptal puncture and screening for peri-device leaks before complete device deployment [16]. A peri-device leak (PDL) > 5 mm was considered clinically relevant. The post-procedural management was directed by the treating physician.

### Data acquisition

This sub-study of the LAARGE registry divided the patient cohort into two groups: patients with prior stroke (stroke group) and patients without prior stroke (non-stroke group). Patient data was collected from each centre through a web-based electronic case report form (CRF). Data was stored in compliance with the German data privacy laws. The collected data included baseline characteristics, medication, imaging and procedural data, complications, and adverse events until hospital discharge. The indication for LAAO was determined by the attending physician. Only those indications should be entered in “other” indications that did not appear to the treating physician to be an absolute contraindication to long-term OAC alone, but which, in combination with other risk factors, were decisive for refraining from OAC and choosing LAAO as an alternative. The follow-up encapsulated data collected at the end of the first year after the LAAO and was carried out by IHF through telephone interviews and by the implantation centres based on a standardized CRF. Relevant details pertaining to clinical status, complications, adverse events, and antithrombotic medication were documented. In the scenario of a potential complication or an adverse event, medical reports pertaining to treatment were collected and reviewed by an independent critical event committee to ensure an unbiased assessment. If patients could not be contacted, information on vital status was obtained from the registration offices.

### Outcome measures

 In this sub-study, the effectiveness of treatment was primarily assessed by the combined absence of all-cause death or non-fatal stroke and, secondarily, by the absence of transient ischemic attack (TIA) or systemic embolism (SE). Concerning the primary effectiveness outcome measure, high-risk patients (male gender, age ≥ 80 years, or estimated glomerular filtration rate (eGFR) ≤ 30 mL/min/1.73 m^2^) were considered separately. Procedural success was defined as stable implantation of an occluder in the left atrial appendage without PDL > 5 mm. The safety of the therapy was assessed with data documenting complications occurring during hospital stay, device-related complications, or those that could be associated with (absence of) antithrombotic therapy during the first year.

### Statistical analyses

Statistical analyses were performed on available data with SAS® version 9.4 (SAS Institute, Cary, NC, USA). Continuous data are presented as means with standard deviation or as medians with interquartile ranges (25^th^ and 75^th^ percentiles), while categorical data as frequencies with group-related percentages. Metric variables were compared with the Mann–Whitney-Wilcoxon test, categorical variables with the Pearson’s chi-squared test, and the Fisher’s exact test in case of low frequencies, respectively. The incidence of the combined event of all-cause death or non-fatal stroke at 1 year post-implantation was evaluated using the Kaplan–Meier estimator and log-rank test. All statistical analyses presented, including *p*-values, have to be interpreted as descriptive. No adjustment for multiple inference and no hierarchical testing has been performed. With these precautions, *p*-values ≤ 0.05 (two-tailed) were considered statistically significant.

## Results

### Baseline characteristics

This study cohort included a total of 638 patients, classified further into a stroke group (*n* = 137; 21.5%) and a non-stroke group (*n* = 501; 78.5%; Table 1). The stroke according to which the group was assigned had occurred at a median of 1 (0; 8) years prior to the index procedure. The representative population was predominantly elderly male with a median age of 76 years (62.8% male) in the stroke group and 77 years (60.7% male) in the non-stroke group. The pattern of AF was almost evenly distributed in both groups, with permanent AF prevalent in 40.9% and 38.5% among the stroke and non-stroke patients groups, respectively (*p* = not significant; NS).

**Table 1:** Baseline characteristics

	Stroke group	Non-stroke group	*p*-value*
Total cohort, *n* (% of all patients)	137 (21.5)	501 (78.5)	-
Male sex, *n* (%)	86 (62.8)	304 (60.7)	0.66
Age [years], median (IQR)	76 (72; 80)	77 (73; 82)	0.09
Body mass index [kg/m^2^], median (IQR)	26.4 (24.1; 29.8)	26.8 (24.2; 30.2)	0.61
CHA_2_DS_2_-VASc score, mean ± SD	5.9 ± 1.3	4.1 ± 1.4	**< 0.001**
HAS-BLED score, mean ± SD	4.6 ± 1.0	3.7 ± 1.1	** < 0.001**
Arterial hypertension, *n* (%)	132 (96.4)	461 (92.0)	0.08
Diabetes mellitus, *n* (%)	53 (38.7)	164 (32.7)	0.19
Congestive heart failure, *n* (%)	33 (24.1)	139 (27.7)	0.39
Coronary heart disease, *n* (%)	58 (42.3)	234 (46.7)	0.36
eGFR [MDRD], median (IQR)	65.9 (41.8; 86.0)	61.0 (41.7; 78.3)	0.17
Pattern of AF, each *n* (%)			
• Paroxysmal	58 (42.3)	216 (43.1)	0.87
• Persistent	23 (16.8)	92 (18.4)	0.67
• Permanent	56 (40.9)	193 (38.5)	0.62
Prior pulmonary vein isolation, *n* (%)	6 (4.4)	11 (2.2)	0.16
Indication for LAAO, each *n* (%)			
• Prior bleeding	97 (70.8)	410 (81.8)	**0.005**
• Contraindication to OAC	28 (20.4)	93 (18.6)	0.62
• Labile INR	12 (8.8)	42 (8.4)	0.89
• OAC incompliance	8 (5.8)	25 (5.0)	0.69
• Patient’s preference	30 (21.9)	131 (26.1)	0.31
• Other reason	15 (10.9)	43 (8.6)	0.39
Ejection fraction [%], median (IQR)	60 (50; 60)	60 (50; 60)	0.75
Ejection fraction ≤ 40%, *n* (%)	14 (10.7)	60 (12.3)	0.61
LA diameter [mm], median (IQR)**	47 (44; 50)	48 (44; 52)	0.223
LA area [cm^2^], median (IQR)**	30 (21; 44)	26 (20; 32)	0.21

^*^ Tested by Pearson’s chi-squared or Mann–Whitney-Wilcoxon test; bold indicates *p *≤ 0.05; more than one item could occur in the same patient; **these two measurements could be documented alternatively; *AF*, atrial fibrillation; *eGFR*, estimated glomerular filtration rate; *INR*, international normalized ratio; *IQR*, interquartile range; *LA(AO)*, left atrial (appendage occlusion); *MDRD*, Modification of Diet in Renal Disease [equation]; *OAC*, oral anticoagulation; *SD*, standard deviation

Multiple indications could be given for LAAO: In 70.8% and 81.8% of cases a bleeding event was reported, respectively (*p* = 0.005). In patients with a history of stroke, the rate of a severe bleeding event was lower as compared to the non-stroke group (67.0% vs. 45.6%, *p* < 0.001). Under “other” indication, the following was stored in aggregated form in the free text entries: adenoma with bleeding risk, amyloid angiopathy, angiodysplasia, apheresis/dialysis therapy, left atrial (appendage) thrombus despite OAC, malassimilation syndrome, mild coagulopathy, mild liver disease, non-bleeding anaemia, recurrent need for additive antiplatelet agent, repeated surgical procedures, tendency to fall, vascular aneurysm. The mean CHA_2_DS_2_-VASc score in the stroke group was 5.9 ± 1.3 points and 4.1 ± 1.4 points in the non-stroke group (*p* < 0.001). Similarly, the risk of bleeding events differed with a mean HAS-BLED score of 4.6 ± 1.0 points and 3.7 ± 1.1, respectively (*p* < 0.001). Clinical parameters and anatomical and functional features derived from pre-procedural cardiac imaging showed no statistically significant difference between the two patient groups.

### Peri-procedural data

The implantation was performed in a total of 137 stroke group patients and 498 non-stroke group patients. Three procedures had to be terminated prematurely in the non-stroke group. A successful implantation was reported in 97.4% of the non-stroke group patients. This was similar to the stroke group with success in 98.5% of the patients (*p* = NS) (Table 2). An intra-procedural PDL > 5 mm was not evident in any of the patients. There were no statistically significant differences in the duration of the procedure or the fluoroscopy time between both groups (*p* = NS). The Watchman™ device (Boston Scientific, Marlborough, MA, USA) and the Amplatzer™ family (Cardiac Plug or Amulet™; Abbott, Chicago, IL, USA) were the most frequently implanted occluders. Patients from the non-stroke group received the Watchman™ device more often (46.8% vs. 32.1%, *p* = 0.002).

**Table 2:** Procedural data

	Stroke group	Non-stroke group	*p*-value*
LAA morphology, each *n* (%)			
• Cactus	13 (10.8)	40 (8.7)	0.46
• Chicken wing	51 (42.5)	209 (45.3)	0.58
• Windsock	18 (15.0)	71 (15.4)	0.91
• Cauliflower	25 (20.8)	65 (14.1)	0.07
• Not determined	13 (10.8)	76 (16.5)	0.13
Successful implantation, *n* (%)	135 (98.5)	488 (97.4)	0.44
Number of implantation attempts, mean ± SD	1.6 ± 1.1	1.6 ± 1.2	0.31
Peri-device leak, *n* (%)	7 (5.3)	25 (5.2)	0.94
• < 3 mm, *n*	4	20	
• 3–5 mm, *n*	3	5	
• > 5 mm, *n*	0	0	
Type of LAAO device, each *n* (%)			
• Watchman™	44 (32.1)	234 (46.8)	**0.002**
• Amplatzer™ cardiac plug	47 (34.3)	130 (26.0)	0.054
• Amplatzer™ Amulet™	41 (29.9)	122 (24.4)	0.19
• Other device	5 (3.6)	14 (2.8)	0.60
Total duration [min], median (IQR)	58 (43; 73)	60 (43; 80)	0.34
Fluoroscopy time [min], median (IQR)	11 (7; 15)	10 (7; 15)	0.56
Dose area product [cGy*cm^2^], median (IQR)	2439 (1158; 4720)	1957 (739; 4081)	**0.015**
Sedation type, each *n* (%)			
• None	1 (0.7)	11 (2.2)	0.26
• Conscious sedation	115 (83.9)	421 (84.2)	0.94
• General anesthesia	16 (11.7)	57 (11.4)	0.93
• Other	6 (4.4)	12 (2.4)	0.22

^*^ Tested by Pearson's chi-squared or Mann–Whitney-Wilcoxon test; *IQR*, interquartile range; *LAA(O)*, left atrial appendage (occlusion); *SD*, standard deviation

Three major adverse cardiac and cerebrovascular events (MACCE) were recorded, all in the non-stroke group (*p* = NS) (Table 3). Twenty-six cases of other major complications were reported (*p* = NS). The combined incidence rate of these events was 4.4% vs. 4.6%, respectively (*p* = NS). Among these, only 1 case of in-hospital stroke occurred in the non-stroke group (*p* = NS). A total of 2.9% of patients from the stroke group developed a pericardial effusion, with 2 of them requiring an intervention. Similarly, 4.4% of the patients from the non-stroke group developed a pericardial effusion and 13 required an intervention/surgery (each *p* = NS). Two patients with no prior history of stroke died during hospital stay; however, these cases of death were not identified to be procedure-related and have been classed as adverse events. The median duration of hospital stay after the procedure was 2 days in both groups.

**Table 3:** Peri-procedural events and intrahospital outcome

	Stroke group	Non-stroke group	*p*-value*
MACCE, *n* (%)	0 (0)	3 (0.6)	1.00
• Death, *n* (%)	0 (0)	2 (0.4)	1.00
• Myocardial infarction, *n* (%)	0 (0)	1 (0.2)	1.00
• Non-fatal stroke, *n* (%)	0 (0)	1 (0.2)	1.00
Other major complications, *n* (%)	6 (4.4)	20 (4.0)	0.81
• Severe bleeding, *n* (%)	1 (0.7)	6 (1.2)	1.00
• AV fistula or pseudoaneurysm, *n* (%)	2 (1.5)	4 (0.8)	0.61
• Pericardial effusion requiring action, each *n* (%)			
• Surgical	0 (0)	2 (0.4)	1.00
• Interventional	2 (1.5)	11 (2.2)	0.74
• Device dislodgement requiring action, each *n* (%)			
• Surgical	0 (0)	0 (0)	-
• Interventional	1 (0.7)	1 (0.2)	0.38
Moderate complications, *n* (%)	12 (8.8)	50 (10.0)	0.75
• Moderate bleeding, *n* (%)	1 (0.7)	11 (2.2)	0.48
• Extended groin haematoma, *n* (%)	3 (2.2)	15 (3.0)	0.78
• Access site infection, *n* (%)	0 (0)	1 (0.2)	1.00
• Pericardial effusion with conservative treatment, *n* (%)	2 (1.5)	9 (1.8)	1.00
• Device dislodgement handled by immediate retraction, *n* (%)	2 (1.5)	5 (1.0)	0.65
• Transient ischemic attack, *n* (%)	0 (0.0)	0 (0.0)	-
• Successful cardiopulmonary resuscitation, *n* (%)	0 (0)	3 (0.6)	1.00
• Other moderate complication, *n* (%)	6 (4.4)	11 (2.2)	0.23
Length of stay after the procedure [d], median (IQR)	2 (2; 3)	2 (2; 4)	0.97

^*^ Tested by Fisher’s exact or Mann–Whitney-Wilcoxon test; more than one item could occur in the same patient; *AV*, arteriovenous; *IQR*, interquartile range; *MACCE*, major adverse cardiac and cerebrovascular event

### Events during follow-up

**Fig. 1:**
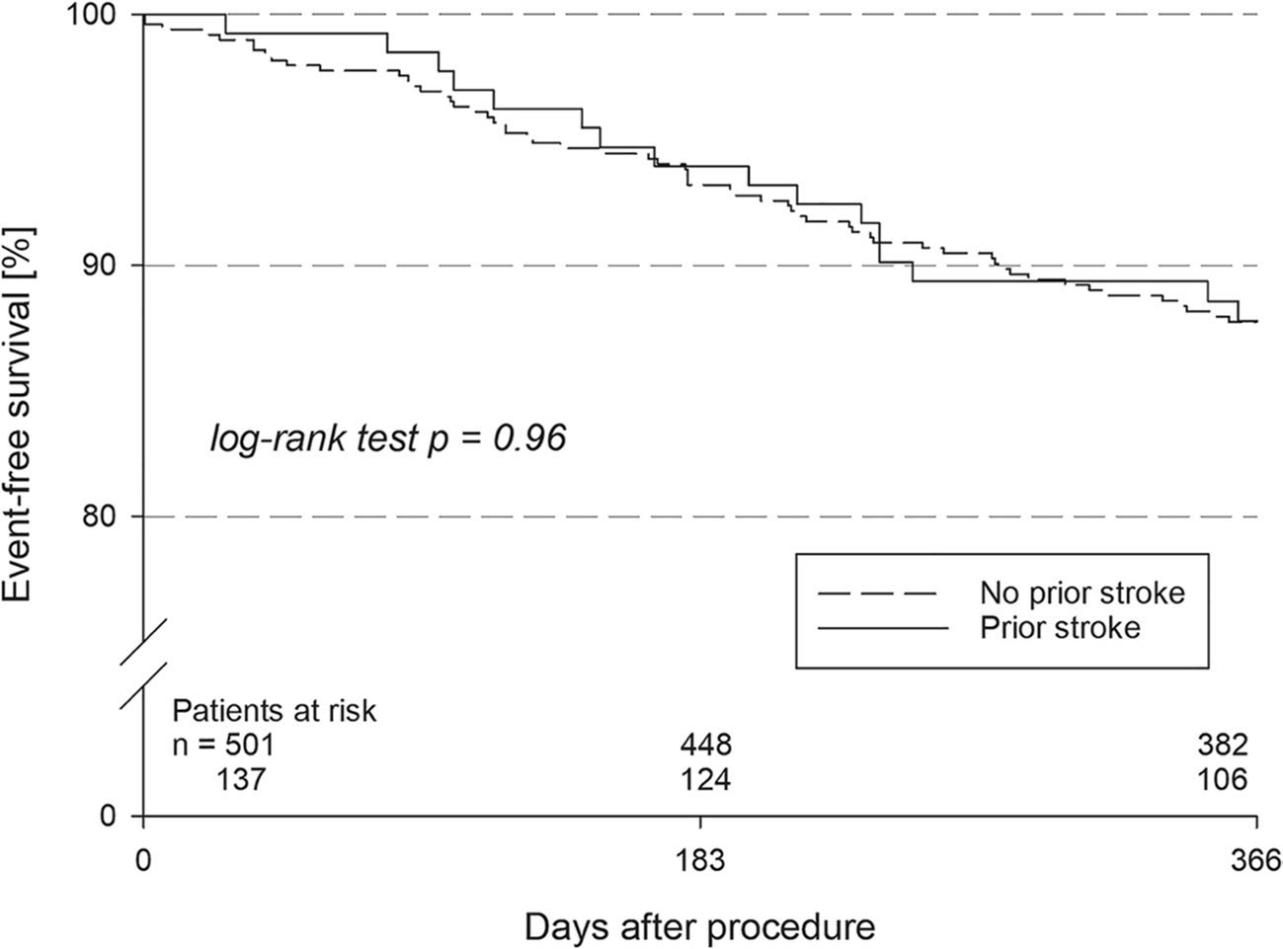
Kaplan–Meier analysis for the combined primary effectiveness outcome measure (absence of all-cause death or non-fatal stroke)

Freedom from all-cause death or non-fatal stroke was not statistically different between both groups (87.8% vs. 87.7% including peri-procedural events; *p* = NS; Fig. 1) The post-discharge 1-year mortality for the stroke group was 10.4%, while it was 11.3% for the non-stroke group (*p* = NS) (Table 4). After hospital discharge, 2 ischemic strokes were documented (1.9%) in the stroke group and 1 case (0.2%) in the non-stroke group (*p* = NS). Two patients (0.5%) from the non-stroke group reported a TIA (*p* = NS). SE occurred in 1 patient in the stroke group and none in the non-stroke group (*p* = NS). When only patients from the stroke group were considered and stratified by stroke on anticoagulation versus stroke without anticoagulation (see the next section for detailed information), there was a trend towards a lower event-free survival (for primary effectiveness outcome measure) in the first group (81.1% vs. 91.5%; *p* = 0.082). This was due to deaths following hospital discharge (16.7 vs. 7.1%; *p* = 0.087). Two strokes after hospital discharge were distributed 1:1 between these two groups. In none of the sub-groups of male patients, age ≥ 80 years or eGFR ≤ 30 mL/min/1.73 m^2^, respectively, the freedom from all-cause death or non-fatal stroke was statistically significantly different between patients with or without prior stroke (Supplementary Table 1). However, patients with each risk factor had a lower event-free rate driven by cases of death.

**Table 4:** Events during 1-year follow-up after hospital discharge

	Stroke group	Non-stroke group	*p*-value*
Discharged alive, *n*	137	499	
Information on vital status obtained, *n* (%)	134 (97.8)	489 (98.0)	0.89
Death within 365 days,* n* (% of patients with documented vital status)	14 (10.4)	55 (11.3)	
Surviving patients with detailed follow-up information	107	404	
Major events – post-discharge in survivors of follow-up			
• Non-fatal stroke, *n* (%)	2 (1.9)	1 (0.2)	0.11
• Transient ischemic attack, *n* (%)	0 (0.0)	2 (0.5)	0.47
• Systemic embolism, *n* (%)	1 (0.9)	0 (0)	0.21
• Severe bleeding, *n* (%)	0 (0)	3 (0.7)	1.00
• Severe groin complication, *n* (%)	0 (0)	1 (0.2)	1.00
• Pericardial effusion requiring action, *n* (%)	0 (0)	1 (0.2)	1.00
• Device dislodgement requiring action, each *n* (%)			
o Surgical	0 (0)	3 (0.6)	1.00
o Interventional	0 (0)	1 (0.2)	1.00
• Pulmonary embolism, *n* (%)	0 (0)	6 (1.5)	0.35
Moderate events – post-discharge in survivors of follow-up			
• Moderate bleeding, *n* (%)	1 (0.9)	19 (4.7)	0.07
• Deep vein thrombosis, *n* (%)	1 (0.9)	1 (0.2)	0.31
Rehospitalisation, *n* (%)	28 (28.9)	147 (40.7)	**0.033**
• Due to an occluder complication, *n* (% of all rehospitalisations)	0 (0)	7 (4.8)	0.23

^*^ Tested by Pearson's chi-squared, Fisher’s exact, or Mann–Whitney-Wilcoxon test; bold indicates *p* ≤ 0.05; more than one item could occur in the same patient

After hospital discharge, an occluder-related complication was reported in 1.8% and 3.3% of patients, respectively (*p* = NS). A moderate or severe bleeding event was reported in 1 patient (0.9%) from the stroke group and 22 patients (5.4%) from the non-stroke group (*p* = 0.062). A similar rate for pericardial effusion at follow-up was also noted (0.9% vs. 1.2%, *p* = NS). Only 1 patient from the non-stroke group required pericardiocentesis. An occluder dislocation at follow-up was seen in 0.9% and 1.2% of the patients, respectively (*p* = NS). A statistically significantly higher number of patients from the non-stroke group were re-admitted to the hospital for various reasons (*p* = 0.033).

### Antithrombotic medication in the stroke patients before procedure

**Fig. 2:**
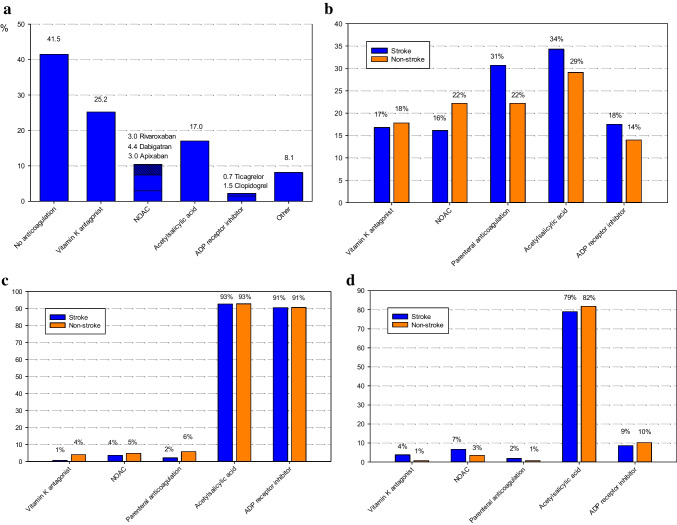
**a** Antithrombotic treatment at the time of the index stroke **b** Antithrombotic treatment on admission **c** Antithrombotic treatment at hospital discharge **d** Antithrombotic treatment at follow-up. Figure caption for the entire Fig. 2: one and the same patient can receive more than one substance; *ADP*, adenosine diphosphate; *NOAC*, non-vitamin K antagonist oral anticoagulant

Patients with a history of stroke received in only 62.0% of cases any form of anticoagulation before the procedure, whereas only 35.6% of patients had received OAC at the time of the index stroke (each *p* = NS). A total of 25.2% of these patients had been treated with an OAC in the form of vitamin K antagonist (VKA) (Fig. 2a and b).

### Antithrombotic medication at hospital discharge and follow-up

The sole use of anticoagulation was recommended in 2.9% of the patients from the stroke group and in 2.8% from the non-stroke group (*p* = NS; Fig. 2). The proportion of patients receiving the combination of OAC and antiplatelet agent(s) was lower in the stroke group than the non-stroke group (3.6% vs. 11.0%; *p* = 0.009). At hospital discharge, a total of 6.6% of the stroke group patients and 13.8% of the non-stroke group were recommended some form of anticoagulation (i.e., OAC, NOAC, unfractionated heparin, or low-molecular-weight heparin; *p* = 0.022).

At follow-up after 1 year, the proportion of patients continuing any antithrombotic medication was high in the stroke as well as non-stroke group (90.5% vs. 88.6%, *p* = NS). The number of patients continuing therapy solely with one or two antiplatelet agent(s) was also not statistically significantly different (78.1% vs. 83.6%; *p* = NS), while the vast majority of these patients were taking mono antiplatelet therapy (92.1%, *p* = NS). More patients in the stroke group received sole anticoagulation (9.5% vs. 3.5%, *p* = 0.009). The number of patients receiving OAC plus antiplatelet agent(s) was not statistically significantly different in the stroke and non-stroke groups (2.9% vs. 1.5%, *p* = NS) (Fig. 2d).

## Discussion

Nonvalvular AF patients with a history of stroke have by definition higher CHA_2_DS_2_-VASc and HAS-BLED scores and are susceptible to recurrent strokes as well as adverse effects related to long-term OAC [2, 17]. To our knowledge, this LAARGE sub-study shows for the first time in a large all-comers clinical registry a similar effectiveness and safety profile for secondary stroke prevention with LAAO in patients with nonvalvular AF and contraindications for long-term OAC. The overall survival and stroke-free survival rates were similar in the stroke as well as non-stroke group of patients. This even applied to patient groups with a pronounced high-risk profile such as male patients, those with advanced age, or advanced renal insufficiency, although, as expected, they had a higher mortality rate compared to patients without such risk factors [18]. Our data suggest that LAAO could be a reasonable therapeutic option for secondary stroke prevention and might therefore have a favourable influence on good clinical advice for these very high-risk patients.

The European Society of Cardiology and the European Stroke Organisation recommend the initiation/resumption of therapeutic anticoagulation with a NOAC no earlier than 2 days after the index stroke. However, recent data from the Early versus Later Anticoagulation for Stroke with Atrial Fibrillation trial show that early initiation of NOAC treatment within 48 h to 7 days, depending on the stroke size, offers an advantage in reducing the incidence of recurrent ischemic stroke, SE, major extracranial hemorrhage, symptomatic intracranial hemorrhage, or vascular death within 30 days [6]. Yet, patients with previous OAC were not included, nor were patients with higher stroke symptom severity on admission. When interpreting the data on treatment safety in these groups, the results should be treated with caution. Data from the earlier RAF-NOACs study have shown that the combined rate of early recurrence or severe bleeding (within 90 days) was nearly 5% in patients treated with NOACs following an acute ischemic stroke [19]. Moreover, results from the WATCH-AF registry revealed that almost one-fourth of patients with stroke associated with AF were not discharged with any therapeutic anticoagulation, resulting in an increased risk of death or recurrent stroke at 12 months [20]. NOAC therapy has been shown to reduce thromboembolic events by 19% in comparison to warfarin and offers a slightly better safety profile, but its use for secondary stroke prevention has been found to be non-inferior in the sub-group of patients with prior stroke or TIA [21]. In the RESTAIC registry, a 17% cumulative incidence for recurrent stroke at 3 years follow-up without any significant difference between VKA and NOAC was reported [22].

Our data revealed that only about one-third of patients in the stroke group were treated specifically with an OAC on admission. This was similar to the non-stroke group among whom 39.9% of the patients received an OAC. Considering the greater risk profile of the stroke patients, this is highly suggestive of a relevant gap in thromboembolic prophylaxis for this group of patients. Another interesting observation can be made after analysis of data from the anticoagulant therapy received by the stroke group of patients at the time of the index stroke. The use of an OAC was documented to be only 35.6% in this group. This further highlights the poor anticoagulant practice in this group of patients, which could have led to the ischemic stroke, thus underscoring the need for an alternative such as LAAO. In addition, patients who suffered a stroke while on anticoagulation tended to be at higher risk of mortality, which led to a trend towards a lower event-free rate in the combined primary endpoint. In turn, the LAAO was also comparably effective in preventing strokes during follow-up in this high-risk sub-group. There are clear indications that patients who suffer an ischemic stroke under anticoagulation are affected by a greater degree of multimorbidity [23, 24], which may well explain the trend towards an increased mortality rate.

In our study, the successful implantation of an LAAO device was reported in 98.5% (of the stroke group patients, which was not significantly different to the non-stroke group with success in 97.4% of the patients). The intrahospital MACCE and combined incidence rate of major procedure-related complications were acceptable in both groups with 0% vs. 0.6% and 4.4% vs. 4.0%, respectively (each *p* = NS) [25]. These results attest to the safety of the procedure in stroke patients. In the PROTECT AF trial, the LAAO with the Watchman™ device reduced the relative risk of the composite end of stroke, SE, or cardiovascular death by about 40% when compared to warfarin [26]. Data from the PREVAIL trial could also demonstrate non-inferiority regarding cardiac embolism > 7 days post-procedure to warfarin treatment, while additionally suggesting relatively infrequent procedural complications associated with LAAO using the Watchman device [27]. The ASAP study included patients with a mean CHA_2_DS_2_-VASc score of 4.4 points in a non-randomised fashion, who received dual-antiplatelet therapy for 6 months, followed by aspirin monotherapy after receiving the Watchman™ device. At follow-up after 14.4 months, the annual ischemic stroke rate was reported to be 1.7% [28]. Our data corroborates the results from these earlier trials. Stroke recurrence after hospital discharge was documented in 1.9% of cases from the stroke group. This was not significantly different to the incidence of stroke among patients from the non-stroke group (0.2%). These figures are quite low considering that a CHA_2_DS_2_-VASc score of 4–5 points translates to an adjusted stroke rate of around 4.0–6.7% per year [29]. This is especially relevant considering that an earlier study reported the annual stroke rate in persistent AF patients with a prior history of ischemic stroke or TIA to be higher than the stroke rate anticipated by the CHA_2_DS_2_-VASc score [30]. However, as our patient cohort includes patients unsuitable for long-term OAC, this dataset is best compared to results from the ACP multicentre registry and the EWOLUTION registry, which yielded similar results [13, 31].

There was also a similar reduction in the number of bleeding events reported during follow-up in both groups of patients. Although stroke patients have a higher bleeding risk than non-stroke patients [32], post-discharge severe bleeding event rates were similarly low in both (0% vs. 0.7%, *p* = NS). LAAO thus appears to demonstrate the potential to achieve a better patient outcome, particularly by comprehensively reducing the rate of major bleeding in this very high-risk patient population [33]. The numerically increased rate of moderate bleeding events in the non-stroke group could probably be attributed to the indication for LAAO (bleeding events to a significantly higher percentage). The 1-year mortality post-discharge was 10.4% in the stroke group and 11.3% in the non-stroke group (*p* = NS). This rate is slightly higher than that reported in the EWOLUTION registry (9.8%) but could be explained by the higher age of our cohort as well as the higher mean HAS-BLED score at baseline (2.3 points in the EWOLUTION registry) [13]. It must be taken into account that LAAO serves in particular to ensure that the remaining time of life is free of disability, as a high percentage of cardioembolic strokes are associated with persistent disability, especially in older patients [10, 11]. Nevertheless, in view of these data, care should be taken when determining the indication to select those patients who will particularly benefit from this invasive procedure in view of a sufficient life expectancy (> 1 year). In older patients, this can be done well in a team with geriatric experts.

An interesting observation in our dataset was the resumption of therapeutic anticoagulation after 1 year in several patients with prior stroke. This was significantly more than patients from the non-stroke group. The exact reason of the readmission could not be obtained from the original data of the LAARGE registry. It could be debated that this therapeutic anticoagulation helped improve the outcome in the stroke group. However, as the total number of patients receiving this treatment was very low, this is quite unlikely.

## Study limitations

These analyses were based on data extracted from the LAARGE registry, an all-comer registry describing a population treated with LAAO, and are associated with inherent limitations pertaining to such data collection. All interventions were performed independent of the study protocol and relied instead on the operator’s discretion as well as relevant treatment guidelines. Participation was voluntary, and the volume of device implantation varied with each centre and operator. On average, the centres recruited for 283 days, so that with a total of 641 patients in the LAARGE study population (for the current analyses, 3 patients had to be excluded due to insufficient data sets regarding a prior stroke), one patient was included every 16 days. Moreover, levels of operator experience varied, but an assessment of the learning curve associated with this intervention could not be interpreted. The difference in the rate of device implantations per manufacturer between both groups could not be elucidated. As there was no obligatory clinical follow-up, underreporting of adverse events may have occurred. A certain diagnostic uncertainty in TIA diagnosis with the possibility of overrepresentation of this entity must be recognised [34]; however, only isolated cases have been reported overall. Although a relevant number of patients were prescribed anticoagulants after hospital discharge, it is unclear if AF was the sole cause. Our registry data is unfortunately limited by information on the dosages of these medications.

## Conclusion

The similarity concerning 1-year effectiveness and safety between the stroke and non-stroke group of patients, reflected in these results, suggests that LAAO plays an equivalent role in the secondary stroke prevention in the nonvalvular AF patient with contraindication for long-term OAC.

## Supplementary Information

Below is the link to the electronic supplementary material.

Supplementary file1 (DOCX 15 KB)

## Data Availability

All data relevant for the interpretation of the results were provided in aggregated form in the article and tables. Requests to access the datasets should be directed to the corresponding author.

## Abbreviations

AF: Atrial fibrillation
CRF: Case report form
eGFR: Estimated glomerular filtration rate
IHF: [Stiftung] Institut für Herzinfarktforschung
LAAO: Left atrial appendage occlusion
LAARGE: Left-Atrium-Appendage occluder Register – GErmany
MACCE: Major adverse cardiac and cerebrovascular events
(N)OAC: (Non-vitamin K antagonist) oral anticoagulant/anticoagulation
NS: Not significant
PDL: Peri-device leak
RCT: Randomised controlled trial
TIA: Transient ischemic attack
SE: Systemic embolism
VKA: Vitamin K antagonist
