# Supplementary material for: LAA occlusion is effective and safe in very high-risk atrial fibrillation patients with prior stroke: results from the multicentre German LAARGE registry
Source: Clin Res Cardiol. 2024 Jan 31;113(10):1451–62. doi: 10.1007/s00392-024-02376-8 (PMC11420338; doi:10.1007/s00392-024-02376-8)
Supplement: Supplementary file 1 — Supplementary file1 (DOCX 15 KB) [file 392_2024_2376_MOESM1_ESM.docx]

| **Supplementary Table 1** | | | | |
| --- | --- | --- | --- | --- |
|  | **N (Stroke + Non-stroke)** | **Stroke group** | **Non-stroke group** | ***p*-value*** |
| **Primary effectiveness outcome measure at one year after procedure, %*** | | | | |
| **Males** | 86 + 304 | 85.0 | 86.0 | 0.86 |
| **Females** | 51 + 197 | 92.2 | 90.5 | 0.70 |
| **Age ≥ 80 years** | 34 + 164 | 84.8 | 83.4 | 0.81 |
| **Age < 80 years** | 103 + 337 | 88.7 | 89.9 | 0.77 |
| **eGFR ≤ 30 mL/min/1.73 m^2^** | 14 + 46 | 71.4 | 80.0 | 0.61 |
| **eGFR > 30 mL/min/1.73 m^2^** | 117 + 446 | 89.3 | 88.6 | 0.84 |
| ** percentages calculated by Kaplan-Meier estimator, p-values by log-rank test; drop-out before one year is evaluated as independent censoring; eGFR = estimated glomerular filtration rate* | | | | |
